# Strain-Gradient Bar-Elastic Substrate Model with Surface-Energy Effect: Virtual-Force Approach

**DOI:** 10.3390/nano12030375

**Published:** 2022-01-24

**Authors:** Suchart Limkatanyu, Worathep Sae-Long, Hamid Mohammad-Sedighi, Jaroon Rungamornrat, Piti Sukontasukkul, Woraphot Prachasaree, Thanongsak Imjai

**Affiliations:** 1 Department of Civil and Environmental Engineering, Faculty of Engineering, Prince of Songkla University, Songkhla 90112, Thailand; suchart.l@psu.ac.th (S.L.); woraphot.p@psu.ac.th (W.P.); 2 Civil Engineering Program, School of Engineering, University of Phayao, Phayao 56000, Thailand; 3 Mechanical Engineering Department, Faculty of Engineering, Shahid Chamran University of Ahvaz, Ahvaz 6135783151, Iran; hmsedighi@gmail.com; 4 Drilling Center of Excellence and Research Center, Shahid Chamran University of Ahvaz, Ahvaz 6135783151, Iran; 5 Applied Mechanics and Structures Research Unit, Department of Civil Engineering, Faculty of Engineering, Chulalongkorn University, Bangkok 10330, Thailand; jaroon.r@chula.ac.th; 6 Construction and Building Materials Research Center, Department of Civil Engineering, King Mongkut’s University of Technology North Bangkok, Bangkok 10800, Thailand; piti.s@eng.kmutnb.ac.th; 7 School of Engineering and Technology, Center of Excellence in Sustainable Disaster Management, Walailak University, Nakhon Si Thammarat 80161, Thailand; thanongsak.im@wu.ac.th

**Keywords:** virtual force principle, nanobar, surface-energy effect, thermodynamics-based strain gradient, elastic substrate media

## Abstract

This paper presents an alternative approach to formulating a rational bar-elastic substrate model with inclusion of small-scale and surface-energy effects. The thermodynamics-based strain gradient model is utilized to account for the small-scale effect (nonlocality) of the bar-bulk material while the Gurtin–Murdoch surface theory is adopted to capture the surface-energy effect. To consider the bar-surrounding substrate interactive mechanism, the Winkler foundation model is called for. The governing differential compatibility equation as well as the consistent end-boundary compatibility conditions are revealed using the virtual force principle and form the core of the model formulation. Within the framework of the virtual force principle, the axial force field serves as the fundamental solution to the governing differential compatibility equation. The problem of a nanowire embedded in an elastic substrate medium is employed as a numerical example to show the accuracy of the proposed bar-elastic substrate model and advantage over its counterpart displacement model. The influences of material nonlocality on both global and local responses are thoroughly discussed in this example.

## 1. Introduction

In recent years, enormous research efforts by scientists and engineers worldwide have been dedicated to the understanding and characterization of the unique responses of micro-sized and nano-sized structures. Their superior mechanical properties have attracted a wide spectrum of novel applications in modern science and technology [1]. Examples of novel devices employing small-sized structures are biosensors [2], piezoelectric actuators [3], nanosensors [4], and gyroscopes [5]. Profound understanding and characterizing mechanical properties of small-sized structures are critical to rational design procedure and performance assessment of these devices during their service life. In addition, nano-sized structures are commonly used as reinforcement components in nanocomposites due to their excellent mechanical properties [6,7]. Generally, the response characteristic of structures at microscale and nanoscale is drastically different from the corresponding response at macroscale due to two unique features inherent to micro-sized and nano-sized structures, namely the small-scale effect and the size-dependent effect. The former effect is related to the discrete nature of matter, thus inducing the material nonlocality while the latter is associated with excessive energy stored in the surface due to high surface-to-volume ratio, hence resulting in the size-dependency characteristic.

Experimental studies and atomistic/molecular dynamic modeling have been carried out by researchers to gain a thorough understanding of mechanical responses of structures at microscale and nanoscale. Due to the small-sized nature of specimens, experimental studies generally require high-precision apparatus and special testing procedure [8]. However, atomistic/molecular dynamic modeling is a viable method to characterize mechanical responses of micro-sized and nano-sized structures and can provide comprehensive simulation information [9,10] but high computational expense must be paid [11]. Consequently, only systems with limited amounts of atoms and molecules can be practically investigated, thus an alternative modeling approach with a superior computational efficiency is deemed essential. Collaboration between a structural-mechanics model (bar, beam, plate, and shell) and non-classical elasticity theory has been carried out by researchers to develop an alternative tool to characterize mechanical responses of small-sized structures [12,13,14,15,16,17,18]. This integrated modeling approach could account for the small-scale effect as well as the size-dependent effect with good balance between accuracy and computational efficiency.

Long-range inter-atomic forces associated with the discrete nature of materials are more influential when the dimension of a structure is in the range spanning from nanoscale to microscale. In the literature, this phenomenon is often referred to as the “small-scale” effect and induces nonlocality in the material. The assertion of material nonlocality is that dependency of a stress at a generic point is not only on the strain at that particular point, but also on those strains and related quantities at all other points throughout the elastic body. Several amended elasticity models have been proposed in the literatures to account for this discrete nature of materials [19,20,21,22,23,24,25,26]. Most widely used among them is the Eringen differential form of the strain-driven nonlocal elasticity model [20,21]. Consequently, a myriad of structural-mechanics models has been armed with this nonlocal constitutive model to account for the small-scale effect [12,27,28,29,30,31,32]. Unfortunately, those enhanced structural-mechanics models usually result in debatable and discrepant responses as pointed out by several researchers [27,33,34]. Romano et al. [35] have thoroughly diagnosed the cause of these problematic responses and concluded that an ill-posed mathematical problem is encountered with the adoption of Eringen nonlocal differential model. In addition, the Eringen nonlocal differential model would not accept quadratic energy functional form of elasticity [36] and the work conjugate nature of stress and strain in this nonlocal constitutive model is ambiguous [37].

Other rational nonlocal constitutive models have been proposed and adopted by various researchers to remedy the debatable and discrepant features inherent to the Eringen nonlocal structural-mechanics models [24,38,39,40,41,42]. Among these rational theories, the thermodynamics-based strain gradient model proposed by Barretta and Marotti de Sciarra [24] is of special interest since it could be adopted with reasonable effort. It is worth mentioning here that nanobars and nanobeams based on this thermodynamics-based strain gradient model do not present debatable and discrepant responses [43,44,45]. Therefore, this study would employ the thermodynamics-based strain gradient model of Barretta and Marotti de Sciarra [24] to represent the material nonlocality.

In opposition to mechanical responses at macroscale, the surface free energy related to surface stress and surface elasticity affects mechanical responses of structures at nanoscale. In literatures, this phenomenon is referred to as the “surface-energy” effect and induces the size dependency of nano-sized structures. To enhance structural-mechanics models with the surface-energy effect, the surface elasticity theory of Gurtin and Murdoch [46,47] has been widely adopted [48,49,50,51,52,53,54]. In this surface elasticity model, the surface layer of a solid core is considered a negligibly thin membrane perfectly bonded to the wrapped solid core.

Nanowires have found a wide spectrum of novel applications in nanoscience and nanotechnology covering optoelectronics, biotechnology, biosensors, and micro/nano electro-mechanical systems (M/NEMS) due to their outstanding mechanical, electrical, and thermal performances [55,56,57,58,59]. In these novel applications, nanowires have often been fabricated into larger parts via polymer substrate media. As a result, the interactive mechanism between the nanowire and its surrounding polymer substrate is of practical value, and plays a crucial role in designing and controlling of performance of devices and systems in such novel applications. In the literature, researchers have developed different nanobeam-elastic substrate models to characterize responses of nanowire-elastic substrate systems. For example, Ponbunyanon et al. [60] analytically investigated static flexural responses of silver nanowire-elastic substrate systems; Zhao et al. [61] analytically conducted buckling load analyses of nanowire-elastic substrate systems; Malekzadeh and Shojaee [62] used beam models to study nonlocal and surface-energy effects on vibrating responses of nanowire-elastic substrate systems.

In the literature, analytical models—though limited in number—have been devoted to characterize the tensile response of nanobar-elastic substrate systems [12,63] and the “irrational” Eringen nonlocal differential model has been employed in those models. Recently, Sae-Long et al. [44] has proposed a rational nanobar-substrate model within the framework of the virtual displacement principle. The thermodynamics-based strain-gradient model of Barretta and Marotti de Sciarra [24] was employed to represent the bulk-material nonlocality. The debatable and discrepant characteristics inherent to the Eringen nonlocal differential model were eliminated in this model. As a counterpart of the nanobar-substrate model proposed by Sae-Long et al. [44], the fundamental interest of this research work is to develop the nanobar-substrate model within the framework of the virtual force principle. The general idea of the model formulation stems from the Eringen’s nonlocal bar-substrate model proposed by Limkatanyu et al. [63] and Eringen’s nonlocal beam-substrate model proposed by Ponbunyanon et al. [60]. To the best knowledge of the authors, this research work presents, for the first time, the formulation of the strain-gradient bar-substrate model within the framework of the virtual force principle and the merit of this formulation framework is discussed. This developing novelty is a more efficient computational platform and is able to remedy several flaws inherent to the standard displacement-based method, as confirmed in the literature [58,61].

Organization of this research work is as follows: first, introductions to thermodynamics-based strain gradient model, surface elasticity model, and Winkler foundation model are briefly presented. The first two models are respectively employed to account for the small-scale and size-dependent effects, while the third is used to represent the interactive mechanism between the bar and its surrounding elastic substrate. Then, the differential equilibrium equation and end-force equilibrium conditions revealed by Sae-Long et al. [44] using the virtual displacement principle are introduced. The system sectional deformation-force (compliance form) relations are subsequently derived. Next, differential compatibility equations, as well as associated classical and non-classical end-displacement compatibility conditions, are consistently derived using the virtual force principle and form the core of the model formulation. The modified Tonti’s diagram is employed to illustrate the problem formulation within the framework of the virtual force principle. Finally, a nanowire-substrate system is employed as a numerical example to show the accuracy of the proposed nanobar-substrate model and to present the advantage over its counterpart proposed by Sae-Long et al. [44]. Both global and local responses of the nanowire-substrate system are thoroughly discussed. The computer software Mathematica [64] is used to perform all symbolic calculations.

## 2. Strain Gradient Bars with Inclusion of Surface-Free Energy

**Figure 1:**
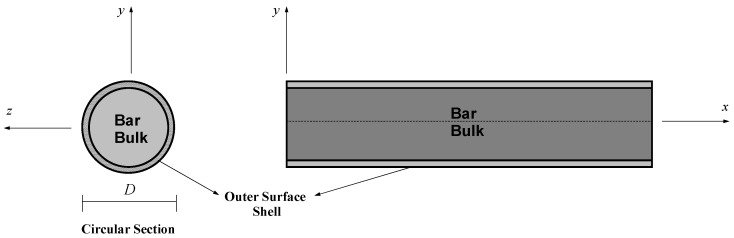
Nanobar section with a warping surface layer.

In the present work, the bar section of Figure 1 is considered a composite composing of a bar-bulk material and a mathematically zero-thickness surface. The simplified strain-gradient elasticity theory of Altan and Aifantis [65] is employed to account for the small-scale effect of the bar-bulk material while the surface elasticity theory of Gurtin and Murdoch [46,47] is used to consider the surface-free energy due to the excess energy at the surface of the bar-bulk material.

### 2.1. Simplified Strain-Gradient Model

As a simple variant of Mindlin’s strain-gradient elasticity theory [19], the simplified strain-gradient elasticity model of Altan and Aifantis [65] is adopted herein to represent higher-order deformation mechanism of materials. This simplified variant is of great interest since it contains only one material small-scale parameter, thus rendering the process of the material-parameter determination and calibration simple and expeditious. Therefore, the simplified strain-gradient elasticity model is well suited to the simplest form of structural-mechanics model-like bars in this study.

For a uniaxial response, the degenerated form of the strain energy density functional Ψ is given by Barretta and Marotti de Sciarra [24] as

(1) $$\Psi \left[\varepsilon_{xx},\eta_{xxx}^{}\right]=\underset{\mathrm{Local}\;\mathrm{term}}{\underset{︸}{\frac{1}{2}E_{xx}{\left(\varepsilon_{xx}\right)}^{2}}}+\underset{\mathrm{Gradient}\;\mathrm{term}}{\underset{︸}{\frac{1}{2}E_{xx}l_{x}{}^{2}{\left(\eta_{xxx}^{}\right)}^{2}}}$$

with $E_{xx}$ being the elastic modulus; $\varepsilon_{xx}\left(x\right)$, the axial strain; $\eta_{xxx}^{}\left(x\right)=\partial \varepsilon_{xx}\left(x\right)/\partial x$, the gradient of axial strain along the *x*-axis (the axial-strain gradient); and $l_{x}$, the material length-scale parameter associated with the axial-strain gradient.

Clearly, the strain energy density functional Ψ of Equation (1) depends not only on the local axial strain $\varepsilon_{xx}\left(x\right)$ but also on the axial-strain gradient $\eta_{xxx}^{}\left(x\right)$, thus inducing nonlocality of the bar-bulk material. Therefore, nonlocality associated with higher-order deformation mechanism of the strain-gradient materials are accounted for through this amended strain energy density functional Ψ.

Following the strain–displacement compatibility relation [24], the axial strain $\varepsilon_{xx}\left(x\right)$ and the axial-strain gradient $\eta_{xxx}^{}\left(x\right)$ can be expressed in terms of the bar axial displacement $u_{x}\left(x\right)$ as

(2) $$\varepsilon_{xx}\left(x\right)=\frac{\partial u_{x}\left(x\right)}{\partial x};\;\;\mathrm{and}\;\;\;\eta_{xxx}^{}\left(x\right)=\frac{\partial^{2}u_{x}\left(x\right)}{\partial x^{2}}$$

To couple the strain energy density functional Ψ with the principle of thermodynamics, the rate form of Equation (1) is required and can be expressed as

(3) $$\dot{\Psi }\left[\varepsilon_{xx},\;\;\eta_{xxx}^{}\right]=\frac{\partial \Psi }{\partial \varepsilon_{xx}}{\dot{\varepsilon }}_{xx}+\frac{\partial \Psi }{\partial \eta_{xxx}^{}}{\dot{\eta }}_{xxx}^{}$$

with (·) denoting the derivative with respect to time t.

Considering the conjugate-work pairs of strain quantities ($\varepsilon_{xx}$ and $\eta_{xxx}^{}$), the stress quantities can be defined based on Equation (3) as

(4) $$\sigma_{xx}^{L}=\frac{\partial \Psi }{\partial \varepsilon_{xx}}=E_{xx}\varepsilon_{xx}\;\;\mathrm{and}\;\;\;\Sigma_{xx}^{}=\frac{\partial \Psi }{\partial \eta_{xxx}^{}}=l_{x}{}^{2}E_{xx}\eta_{xxx}^{}$$

where $\sigma_{xx}^{L}$ defines the local axial stress and represents the conjugate-work pair of the axial strain $\varepsilon_{xx}$; and $\Sigma_{xx}^{}$ defines the higher-order axial stress and represents the conjugate-work pair of the axial-strain gradient $\eta_{xxx}^{}$.

Recalling the first law of thermodynamics, the following expression must be satisfied

(5) $$\int_{L}\left(\int_{A}\sigma_{xx}{\dot{\varepsilon }}_{xx}dA\right)\;dx-\int_{L}\left(\int_{A}\dot{\Psi }\;dA\right)\;dx=0$$

where $\sigma_{xx}$ is the nonlocal axial stress; A, the bar cross-section area; and L, the bar length.

Substituting Equation (3) into Equation (5) leads to the following expression

(6) $$\int_{L}N\left(x\right)\;{\dot{\varepsilon }}_{xx}\;dx-\int_{L}N_{L}\left(x\right)\;{\dot{\varepsilon }}_{xx}dx-\int_{L}N_{H}\left(x\right)\;{\dot{\eta }}_{xxx}^{}dx=0$$

where the sectional resultant forces ($N\left(x\right)$, $N_{L}\left(x\right)$, and $N_{H}\left(x\right)$) are defined as

(7) $$\left[N\left(x\right),\;N_{L}\left(x\right),\;N_{H}\left(x\right)\right]=\int_{A}\left[\sigma_{xx}^{},\;\sigma_{xx}^{L},\;\Sigma_{xx}^{}\right]\;dA\;$$

As demonstrated by Sae-Long et al. [44], the integral relation of Equation (6) plays an essential role in deriving the governing differential equilibrium equation of the nanobar-elastic substrate system via the virtual displacement principle.

### 2.2. Surface Elasticity Theory

The size-dependent phenomenon is unique to nano-sized structures. The so-called “surface-free energy” related to excessive energy at the surface atoms is responsible for this unique phenomenon. This study employs the surface elasticity model of Gurtin and Murdoch [46,47] to account for the surface-energy effect on nano-sized bar responses. For the present problem of nanobars, the degenerated form of the Gurtin–Murdoch surface constitutive model can be written as

(8) $$\tau_{xx}^{sur}-\tau_{0}^{sur}=E^{sur}\varepsilon_{xx}^{sur}$$

where $\tau_{xx}^{sur}$ is the axial component of the surface stress tensor; $\tau_{0}^{sur}$, the residual surface stress under unconstrained conditions; $E^{sur}$, the surface elastic modulus; $u_{xx}^{sur}$, the surface axial displacement; and $\varepsilon_{xx}^{sur}=\partial u_{x}^{sur}/\partial x$, the surface strain.

Following the perfectly bonded interface between the bar bulk and the wrapped surface layer (full composite action), the following relations are obtained

(9) $$u_{xx}^{sur}\left(x\right)=u_{x}\left(x\right)\;\;\mathrm{and}\;\;\;\varepsilon_{xx}^{sur}\left(x\right)=\varepsilon_{xx}\left(x\right)$$

## 3. Bar-Substrate Medium Interaction

To consider interactive mechanism between the bar and its surrounding substrate medium, the widely used Winkler foundation model [66] is called for. Smeared elastic springs in a series are distributed along the bar length to represent the surrounding substrate medium. The force–deformation relation for these smeared elastic springs is

(10) $$D_{S}\left(x\right)=k_{S}\Delta_{S}\left(x\right)$$

with $D_{S}\left(x\right)$ being the substrate interactive force; $k_{S}$, the elastic substrate stiffness; and $\Delta_{S}\left(x\right)$, the substrate deformation.

Full compatibility between the bar and its surrounding substrate medium results in the following relation

(11) $$\Delta_{S}\left(x\right)=u_{x}\left(x\right)$$

## 4. Model Formulation

### 4.1. Differential Equilibrium Equation and End-Force Equilibrium Conditions: The Virtual Displacement Approach

As an alternative to represent the system equilibrium, the virtual displacement principle is called for. The general expression of the virtual displacement principle is

(12) $$\delta W=\delta W_{\mathrm{int}}+\delta W_{\mathrm{ext}}=0$$

where δW represents the system total virtual work; $\delta W_{\mathrm{int}}$ represents the system internal virtual work; and $\delta W_{\mathrm{ext}}$ represents the system external virtual work.

**Figure 2:**
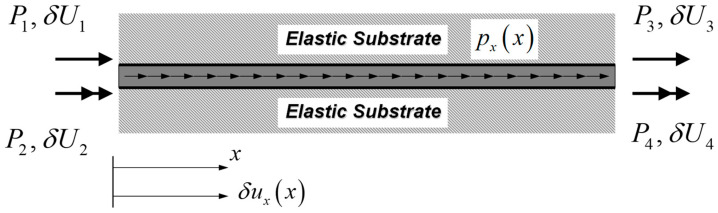
Nanobar-elastic substrate system: the virtual-displacement formulation.

For a strain gradient bar-elastic substrate medium system with inclusion of surface-free energy shown in Figure 2, $\delta W_{\mathrm{int}}$ and $\delta W_{\mathrm{ext}}$ are given by Sae-Long et al. [44] as

(13) $$\begin{array}{l} \delta W_{\mathrm{int}}=\underset{\mathrm{Bar}‐\mathrm{Bulk}\;\mathrm{Contribution}}{\underset{︸}{\int_{L}\left(\int_{A}\sigma_{xx}\left(x\right)dA\right)\delta \varepsilon_{xx}\left(x\right)dx}}+\underset{\mathrm{Substrate}\;-\mathrm{Medium}\;\;\mathrm{Contribution}}{\underset{︸}{\int_{L}D_{S}\left(x\right)\delta \Delta_{S}\left(x\right)dx}}\\ \;\;\;\;\;\;+\;\underset{\mathrm{Surface}\;-\mathrm{Energy}\;\;\mathrm{Contribution}}{\underset{︸}{\int_{L}\left(\oint_{\Gamma }\left(\left(\tau_{xx}^{sur}\left(x\right)-\tau_{0}^{sur}\right)\;\;\delta \varepsilon_{xx}^{sur}\left(x\right)\right)d\Gamma \right)dx}} \end{array}$$

(14) $$\delta W_{\mathrm{ext}}=-\underset{\mathrm{Distributed}‐\mathrm{Load}\;\mathrm{Contribution}}{\underset{︸}{\int_{L}p_{x}\left(x\right)\delta u_{x}\left(x\right)dx}}\;-\underset{\mathrm{End}‐\mathrm{Load}\;\mathrm{Contribution}}{\underset{︸}{\delta U^{T}P}}$$

where Γ is the bar perimeter; $p_{x}\left(x\right)$ represents the longitudinal distributed load; the vector $U={\left\{\begin{array}{cccc} U_{1} & U_{2} & U_{3} & U_{4} \end{array}\right\}}^{T}$ collects displacements at the bar ends; and the vector $P={\left\{\begin{array}{cccc} P_{1} & P_{2} & P_{3} & P_{4} \end{array}\right\}}^{T}$ collects conjugate-work forces at the bar ends.

Recalling the definition of the sectional resultant forces of Equation (7), imposing the compatibility conditions of Equations (2), (9) and (11) and subsequently imposing the thermodynamics condition of Equation (6), the system internal virtual work $\delta W_{\mathrm{int}}$ becomes

(15) $$\begin{array}{l} \delta W_{\mathrm{int}}=\int_{L}N_{L}\left(x\right)\frac{\partial \delta u_{x}\left(x\right)}{\partial x}dx+\int_{L}N_{H}\left(x\right)\frac{\partial^{2}\delta u_{x}\left(x\right)}{\partial x^{2}}dx+\int_{L}D_{S}\left(x\right)\delta u_{x}\left(x\right)dx\\ \;\;\;\;\;\;+\int_{L}N_{\tau_{xx}-\tau_{0}}^{sur}\left(x\right)\frac{\partial \delta u_{x}\left(x\right)}{\partial x}dx \end{array}$$

where the surface axial force $N_{\tau_{xx}-\tau_{0}}^{sur}\left(x\right)$ is defined as

(16) $$N_{\tau_{xx}-\tau_{0}}^{sur}\left(x\right)=\oint_{\Gamma }\left(\tau_{xx}^{sur}\left(x\right)-\tau_{0}^{sur}\right)\;\;d\Gamma$$

Consequently, the virtual displacement statement of Equation (12) is rewritten as

(17) $$\begin{array}{l} \delta W=\int_{L}N_{L}^{sur}\left(x\right)\frac{\partial \delta u_{x}\left(x\right)}{\partial x}dx+\int_{L}N_{H}\left(x\right)\frac{\partial^{2}\delta u_{x}\left(x\right)}{\partial x^{2}}dx+\int_{L}D_{S}\left(x\right)\delta u_{x}\left(x\right)dx\\ \;\;\;\;-\int_{L}p_{x}\left(x\right)\delta u_{x}\left(x\right)dx-\delta U^{T}P=0 \end{array}$$

where $N_{L}^{sur}\left(x\right)=N_{L}\left(x\right)+N_{\tau_{xx}-\tau_{0}}^{sur}\left(x\right)$ is defined as the lower-order composite axial force and is contributed from the full composite action between the bar-bulk material and the wrapped surface layer.

Following the virtual displacement principle employed by Sae-Long et al. [44], the governing differential equilibrium equation (Euler–Lagrange equation) as well as its associated end-boundary force conditions (natural boundary conditions) of the nanobar-elastic substrate system are consistently derived as

(18) $$\frac{\partial^{2}N_{H}\left(x\right)}{\partial x^{2}}-\frac{\partial N_{L}^{sur}\left(x\right)}{\partial x}+D_{S}\left(x\right)-p_{x}\left(x\right)=0:\;\mathrm{for}\;x\in \left(0,\;L\right)$$

(19) $$\begin{array}{l} P_{1}=-{\left(N_{L}^{sur}\left(x\right)-\frac{\partial N_{H}\left(x\right)}{\partial x}\right)}_{x=0};\;P_{2}=-{\left(N_{H}\left(x\right)\right)}_{x=0};\\ P_{3}={\left(N_{L}^{sur}\left(x\right)-\frac{\partial N_{H}\left(x\right)}{\partial x}\right)}_{x=L};\;P_{4}={\left(N_{H}\left(x\right)\right)}_{x=L} \end{array}$$

It is worth remarking that the differential equilibrium equation of Equation (18) is of vital importance when the virtual force principle is employed to reveal the differential compatibility equation of the problem, as will be presented subsequently.

### 4.2. Sectional Constitutive Relations: Compliance Form

The sectional constitutive relations can be obtained by substituting the stress–strain relations of Equations (4) and (8) into Equations (7) and (16), respectively, and can be written in the compliance form as

(20) $$\varepsilon_{xx}\left(x\right)=\frac{N_{L}\left(x\right)}{E_{xx}A};\;\;\eta_{xxx}\left(x\right)=\frac{N_{H}\left(x\right)}{l_{x}{}^{2}E_{xx}A};\;\mathrm{and}\;\varepsilon_{xx}^{sur}\left(x\right)=\frac{N_{\tau_{xx}-\tau_{0}}^{sur}\left(x\right)}{E^{sur}\Gamma }$$

Imposing the full composite action between the bar bulk and the wrapped surface layer of Equations (9) and (20) provides the following relations between axial force components

(21) $$\begin{array}{l} N_{L}^{}\left(x\right)=\frac{E_{xx}A}{E_{xx}A+E^{sur}\Gamma }N_{L}^{sur}\left(x\right);\;N_{\tau_{xx}-\tau_{0}}^{sur}\left(x\right)=\frac{E^{sur}\Gamma }{E_{xx}A+E^{sur}\Gamma }N_{L}^{sur}\left(x\right);\;\mathrm{and}\\ \;\;\;\;\;\;\;\;\;N_{H}^{}\left(x\right)=\frac{l_{x}{}^{2}E_{xx}A}{E_{xx}A+E^{sur}\Gamma }\frac{\partial N_{L}^{sur}\left(x\right)}{\partial x} \end{array}$$

Based on Equation (10), the deformation-force (compliance form) relation for an elastic substrate medium can be expressed as

(22) $$\Delta_{S}\left(x\right)=\frac{D_{S}\left(x\right)}{k_{S}}$$

### 4.3. Differential Compatibility Equations and End-Displacement Compatibility Conditions: The Virtual Force Approach

To express the system compatibility conditions in the integral (weak) form, the virtual force principle is applied. The virtual force equation can be written in a general form as

(23) $$\delta W^{*}=\delta W_{\mathrm{int}}^{*}+\delta W_{\mathrm{ext}}^{*}=0$$

where $\delta W^{*}$ represents the system total complementary virtual work; $\delta W_{int}^{*}$ represents the system internal complementary virtual work; and $\delta W_{\mathrm{ext}}^{*}$ represents the system external complementary virtual work.

**Figure 3:**
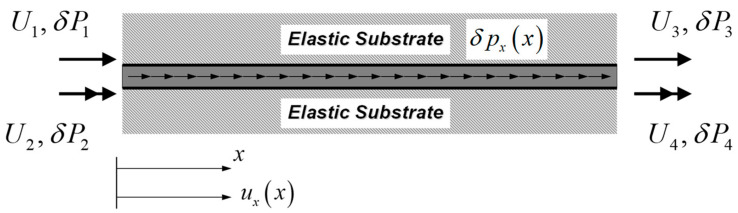
Nanobar-elastic substrate system: the virtual-force formulation.

For the bar-substrate medium system of Figure 3, $\delta W_{\mathrm{int}}^{*}$ and $\delta W_{\mathrm{ext}}^{*}$ are

(24) $$\begin{array}{l} \delta W_{\mathrm{int}}^{*}=\int_{L}\delta N_{L}\left(x\right)\varepsilon_{xx}\left(x\right)\;dx+\int_{L}\delta N_{H}\left(x\right)\eta_{xxx}\left(x\right)\;dx\\ \;\;\;\;\;\;\;+\int_{L}\delta N_{\tau_{xx}-\tau_{0}}^{sur}\left(x\right)\varepsilon_{xx}^{sur}\left(x\right)dx\;\;+\int_{L}\delta D_{S}\left(x\right)\Delta_{S}\left(x\right)\;dx \end{array}$$

(25) $$\delta W_{\mathrm{ext}}^{*}=-\int_{L}\delta p_{x}\left(x\right)u_{x}\left(x\right)dx-\delta P^{T}U$$

To eliminate the bar axial displacement $u_{x}\left(x\right)$ from the virtual force statement, the virtual longitudinal distributed load $\delta p_{x}\left(x\right)$ can arbitrarily be chosen to be zero without loss of model generality. Therefore, Equation (23) becomes

(26) $$\begin{array}{l} \int_{L}\delta N_{L}\left(x\right)\varepsilon_{xx}\left(x\right)\;dx+\int_{L}\delta N_{H}\left(x\right)\eta_{xxx}\left(x\right)\;dx+\\ \;\int_{L}\delta D_{S}\left(x\right)\Delta_{S}\left(x\right)\;dx\;\;+\int_{L}\delta N_{\tau_{xx}-\tau_{0}}^{sur}\left(x\right)\varepsilon_{xx}^{sur}\left(x\right)dx-\delta P^{T}U=0 \end{array}$$

Enforcing the compliance-type constitutive relations of Equations (20) and (22), Equation (26) can be rewritten as

(27) $$\begin{array}{l} \int_{L}\delta N_{L}^{sur}\left(x\right)\frac{N_{L}\left(x\right)}{E_{xx}A}\;dx+\int_{L}\delta N_{H}\left(x\right)\frac{N_{H}\left(x\right)}{l_{x}{}^{2}E_{xx}A}\;dx+\\ \int_{L}\delta D_{S}\left(x\right)\frac{D_{S}\left(x\right)}{k_{S}}\;dx-\delta P^{T}U=0 \end{array}$$

Imposing the differential equilibrium relation of Equation (18), the substrate interactive force $D_{S}\left(x\right)$ and its virtual counterpart $\delta D_{S}\left(x\right)$ can be excluded from the virtual force statement. Thus, Equation (24) is rewritten as

(28) $$\begin{array}{l} \int_{L}\delta N_{L}^{sur}\left(x\right)\frac{N_{L}\left(x\right)}{E_{xx}A}\;dx+\int_{L}\delta N_{H}\left(x\right)\frac{N_{H}\left(x\right)}{l_{x}{}^{2}E_{xx}A}\;dx\\ +\;\int_{L}\left(-\frac{\partial^{2}\delta N_{H}\left(x\right)}{\partial x^{2}}+\frac{\partial \delta N_{L}^{sur}\left(x\right)}{\partial x}\right)\left(\frac{1}{k_{S}}\right)\left(-\frac{\partial^{2}N_{H}\left(x\right)}{\partial x^{2}}+\frac{\partial N_{L}^{sur}\left(x\right)}{\partial x}+p_{x}\left(x\right)\right)\;dx\\ -\;\delta P^{T}U=0 \end{array}$$

In order to move differential operators to axial forces $N_{L}^{sur}\left(x\right)$ and $N_{H}\left(x\right)$, integration by parts is called for, thus resulting in the following expression

(29) $$\begin{array}{cc} \; & \int_{L}\delta N_{L}^{sur}\left(x\right)\left(\frac{N_{L}\left(x\right)}{E_{xx}A}+\left(\frac{1}{k_{S}}\right)\left(\frac{\partial^{3}N_{H}\left(x\right)}{\partial x^{3}}-\frac{\partial^{2}N_{L}^{sur}\left(x\right)}{\partial x^{2}}-\frac{\partial p_{x}\left(x\right)}{\partial x}\right)\right)\;dx+\\ \; & \int_{L}\delta N_{H}\left(x\right)\left(\frac{N_{H}\left(x\right)}{{l_{x}}^{2}E_{xx}A}+\left(\frac{1}{k_{S}}\right)\left(\frac{\partial^{4}N_{H}\left(x\right)}{\partial x^{4}}-\frac{\partial^{3}N_{L}^{sur}\left(x\right)}{\partial x^{3}}-\frac{\partial^{2}p_{x}\left(x\right)}{\partial x^{2}}\right)\right)\;dx+\\ \; & {\left[\frac{1}{k_{S}}\left(-\frac{\partial^{2}N_{H}\left(x\right)}{\partial x^{2}}+\frac{\partial N_{L}^{sur}\left(x\right)}{\partial x}+p_{x}\left(x\right)\right)\left(\delta N_{L}^{sur}\left(x\right)-\frac{\partial \delta N_{H}\left(x\right)}{\partial x}\right)\right]}_{0}^{L}+\\ \; & {\left[\frac{1}{k_{S}}\left(-\frac{\partial^{3}N_{H}\left(x\right)}{\partial x^{3}}+\frac{\partial^{2}N_{L}^{sur}\left(x\right)}{\partial x^{2}}+\frac{\partial p_{x}\left(x\right)}{\partial x}\right)\delta N_{H}\left(x\right)\right]}_{0}^{L}-\delta P^{T}U=0 \end{array}$$

The virtual force quantities in the first boundary term of Equation (29) reveal that the total lower-order (local) axial force $N\left(x\right)$ is defined in terms of the lower-order composite axial force $N_{L}^{sur}\left(x\right)$ and the higher-order axial force $N_{H}\left(x\right)$ as

(30) $$N\left(x\right)\;=\;N_{L}^{sur}\left(x\right)-\frac{\partial N_{H}\left(x\right)}{\partial x}$$

The axial-force relation of Equation (30) was also gained by Sae-Long et al. [44] using the virtual displacement principle as shown in Equation (19). 

Following the Cartesian sign convention and recalling the axial-force definition of Equation (30), Equation (29) becomes

(31) $$\begin{array}{cc} \; & \int_{L}\delta N_{L}^{sur}\left(x\right)\left(\frac{N_{L}\left(x\right)}{E_{xx}A}+\left(\frac{1}{k_{S}}\right)\left(\frac{\partial^{3}N_{H}\left(x\right)}{\partial x^{3}}-\frac{\partial^{2}N_{L}^{sur}\left(x\right)}{\partial x^{2}}-\frac{\partial p_{x}\left(x\right)}{\partial x}\right)\right)\;dx+\\ \; & \int_{L}\delta N_{H}\left(x\right)\left(\frac{N_{H}\left(x\right)}{{l_{x}}^{2}E_{xx}A}+\left(\frac{1}{k_{S}}\right)\left(\frac{\partial^{4}N_{H}\left(x\right)}{\partial x^{4}}-\frac{\partial^{3}N_{L}^{sur}\left(x\right)}{\partial x^{3}}-\frac{\partial^{2}p_{x}\left(x\right)}{\partial x^{2}}\right)\right)\;dx+\\ \; & -\delta P_{1}\left(U_{1}+\frac{1}{k_{S}}{\left(-\frac{\partial^{2}N_{H}\left(x\right)}{\partial x^{2}}+\frac{\partial N_{L}^{sur}\left(x\right)}{\partial x}+p_{x}\left(x\right)\right)}_{x=0}\right)\\ \; & -\delta P_{2}\left(U_{2}-\frac{1}{k_{S}}{\left(-\frac{\partial^{3}N_{H}\left(x\right)}{\partial x^{3}}+\frac{\partial^{2}N_{L}^{sur}\left(x\right)}{\partial x^{2}}+\frac{\partial p_{x}\left(x\right)}{\partial x}\right)}_{x=0}\right)\\ \; & -\delta P_{3}\left(U_{3}+\frac{1}{k_{S}}{\left(-\frac{\partial^{2}N_{H}\left(x\right)}{\partial x^{2}}+\frac{\partial N_{L}^{sur}\left(x\right)}{\partial x}+p_{x}\left(x\right)\right)}_{x=L}\right)\\ \; & -\delta P_{4}\left(U_{4}-\frac{1}{k_{S}}{\left(-\frac{\partial^{3}N_{H}\left(x\right)}{\partial x^{3}}+\frac{\partial^{2}N_{L}^{sur}\left(x\right)}{\partial x^{2}}+\frac{\partial p_{x}\left(x\right)}{\partial x}\right)}_{x=L}\right)=0 \end{array}$$

Accounting for arbitrariness of $\delta N_{L}^{sur}\left(x\right)$ and $\delta N_{H}\left(x\right)$, the governing differential compatibility equations associated with the lower-order and higher-order axial forces are obtained, respectively, as

(32) $$\frac{N_{L}\left(x\right)}{E_{xx}A}+\left(\frac{1}{k_{S}}\right)\left(\frac{\partial^{3}N_{H}\left(x\right)}{\partial x^{3}}-\frac{\partial^{2}N_{L}^{sur}\left(x\right)}{\partial x^{2}}-\frac{\partial p_{x}\left(x\right)}{\partial x}\right)=0:\;\mathrm{for}\;x\in \left(0,\;L\right)$$

(33) $$\frac{N_{H}\left(x\right)}{l_{x}{}^{2}E_{xx}A}+\left(\frac{1}{k_{S}}\right)\left(\frac{\partial^{4}N_{H}\left(x\right)}{\partial x^{4}}-\frac{\partial^{3}N_{L}^{sur}\left(x\right)}{\partial x^{3}}-\frac{\partial^{2}p_{x}\left(x\right)}{\partial x^{2}}\right)=0:\;\mathrm{for}\;x\in \left(0,\;L\right)$$

It is worth remarking that due to elimination of the substrate interactive force $D_{S}\left(x\right)$ and its virtual counterpart $\delta D_{S}\left(x\right)$ as discussed earlier, the compatibility condition associated with the elastic-substrate medium is not present in the virtual force statement of Equation (31). 

Considering the compliance-type constitutive relations of Equations (20) and (22), enforcing the Winkler-foundation assumption of Equation (11), and imposing the equilibrium relation of Equation (18), Equations (20) and (22) simply address the lower-order and higher-order strain–displacement compatibility conditions as

(34) $$\varepsilon_{xx}\left(x\right)-\frac{\partial u_{x}\left(x\right)}{\partial x}=0$$

(35) $$\eta_{xxx}^{}\left(x\right)-\frac{\partial^{2}u_{x}\left(x\right)}{\partial x^{2}}=0$$

Considering the first and third axial-force relations of Equation (21), the following relation between the first derivative of the local axial force $N_{L}\left(x\right)$ and higher-order axial force $N_{H}\left(x\right)$ can be established by

(36) $$N_{H}\left(x\right)=l_{x}{}^{2}\frac{\partial N_{L}\left(x\right)}{\partial x}$$

With the axial-force relation of Equation (36), two differential compatibility conditions of Equations (32) and (33) can be combined into a single expression as

(37) $$\frac{N_{L}\left(x\right)}{E_{xx}A}+\left(\frac{1}{k_{S}}\right)\left(l_{x}{}^{2}\frac{\partial^{4}N_{H}\left(x\right)}{\partial x^{4}}-\frac{\partial^{2}N_{L}^{sur}\left(x\right)}{\partial x^{2}}-\frac{\partial p_{x}\left(x\right)}{\partial x}\right)=0:\;\mathrm{for}\;x\in \left(0,\;L\right)$$

It is worth restating that the differential equilibrium equation given by Sae-Long et al. [44] is derived based on the virtual displacement principle while the differential compatibility equation of Equation (37) is derived based on the virtual force principle. Comparison between these two differential equations confirms the dualism of the virtual displacement and virtual force principles.

Accounting for the arbitrariness of δP in Equation (31) yields the following end-boundary displacement conditions (essential boundary conditions).

(38) $$\begin{array}{c} U_{1}=\frac{1}{k_{S}}{\left(\frac{\partial^{2}N_{H}\left(x\right)}{\partial x^{2}}-\frac{\partial N_{L}^{sur}\left(x\right)}{\partial x}\right)}_{x=0}-\frac{1}{k_{S}}{\left(p_{x}\left(x\right)\right)}_{x=0}\\ U_{2}=\frac{1}{k_{S}}{\left(-\frac{\partial^{3}N_{H}\left(x\right)}{\partial x^{3}}+\frac{\partial^{2}N_{L}^{sur}\left(x\right)}{\partial x^{2}}\right)}_{x=0}+\frac{1}{k_{S}}{\left(\frac{\partial p_{x}\left(x\right)}{\partial x}\right)}_{x=0}\\ U_{3}=\frac{1}{k_{S}}{\left(\frac{\partial^{2}N_{H}\left(x\right)}{\partial x^{2}}-\frac{\partial N_{L}^{sur}\left(x\right)}{\partial x}\right)}_{x=L}-\frac{1}{k_{S}}{\left(p_{x}\left(x\right)\right)}_{x=L}\\ U_{4}=\frac{1}{k_{S}}{\left(-\frac{\partial^{3}N_{H}\left(x\right)}{\partial x^{3}}+\frac{\partial^{2}N_{L}^{sur}\left(x\right)}{\partial x^{2}}\right)}_{x=L}+\frac{1}{k_{S}}{\left(\frac{\partial p_{x}\left(x\right)}{\partial x}\right)}_{x=L} \end{array}$$

Compared to the end-boundary force conditions given by Sae-Long et al. [44] using the virtual displacement principle, Equation (38) and those given by Sae-Long et al. [44] are dual. Furthermore, end-displacement components associated with the bar bulk-surface layer composite ($N_{L}^{sur}\left(x\right)$ and $N_{H}\left(x\right)$) and the distributed load $p_{x}\left(x\right)$ are clearly separated in Equation (38).

**Figure 4:**
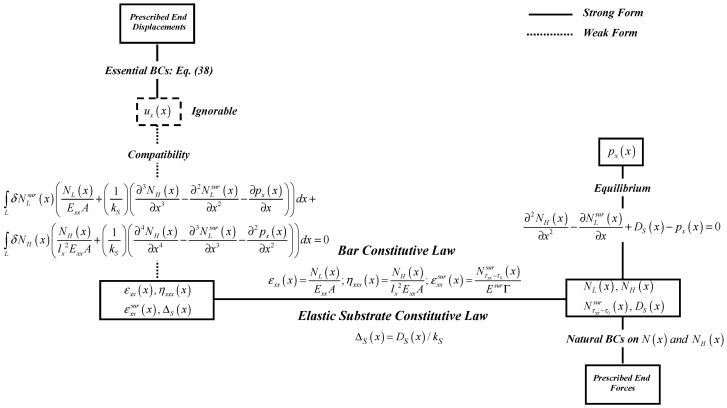
Modified Tonti’s diagram for nanobar-elastic substrate system: The virtual-force formulation.

In summary, a complete set of governing equations of the problem formulated within the framework of virtual force principle are the equilibrium condition of Equation (18), the compliance-type constitutive relations of Equations (20) and (22), and the virtual-force statement (weak form) of the compatibility relations of Equations (32) and (33) together with the end-boundary displacement conditions of Equation (38). The formulation procedure within the framework of the virtual force principle can concisely be presented in the modified Tonti’s diagram of Figure 4.

## 5. Analytical Solution of Differential Compatibility Equation: Axial-Force Solution

Within the framework of the virtual force formulation, the analytical solution to the differential compatibility equation of Equation (37) is expressed in terms of axial force variables. Unfortunately, the present form of Equation (37) cannot be solved readily since it contains multi axial-force variables ($N_{L}\left(x\right)$, $N_{L}^{sur}\left(x\right)$ and $N_{H}\left(x\right)$). Recalling the axial-force relations of Equation (21), the differential compatibility relation of Equation (37) can be expressed in terms of a single axial-force field $N_{L}^{sur}\left(x\right)$ as

(39) $$\left(l_{x}{}^{2}E_{xx}A\right)\frac{\partial^{4}N_{L}^{sur}\left(x\right)}{\partial x^{4}}-{\left(EA\right)}_{xx}^{sur}\frac{\partial^{2}N_{L}^{sur}\left(x\right)}{\partial x^{2}}+\;k_{S}N_{L}^{sur}\left(x\right)={\left(EA\right)}_{xx}^{sur}\frac{\partial p_{x}\left(x\right)}{\partial x}\;\mathrm{for}\;x\in \left(0,\;L\right)$$

with the composite bar axial stiffness ${\left(EA\right)}_{xx}^{sur}$ being defined as $E_{xx}A+E^{sur}\Gamma$.

Equation (39) is central to the axial-force determination of the strain-gradient bar-elastic substrate system with inclusion of surface-energy effect. The strain-gradient nature of the bar-bulk material induces the higher-order derivative (fourth order) while the surface-free energy induces the lower-order derivative (second order). Furthermore, it is observed from Equation (39) that the surface-energy effect influences both homogeneous and particular solutions while the strain-gradient effect only affects the homogeneous solution. It is worth pointing out that with the presence of a uniformly distributed load $p_{x}\left(x\right)=p_{x0}$, only the homogeneous solution is required since the term on the right-hand side of Equation (39) vanishes. In other words, the axial-force response $N_{L}^{sur}\left(x\right)$ is not influenced with the presence of a uniformly distributed load $p_{x}\left(x\right)=p_{x0}$. This unique feature makes the proposed bar-elastic substrate model desirable since there is no need for the particular solution with this specific loading case. However, the presence of the uniformly distributed load $p_{x}\left(x\right)=p_{x0}$ affects the system response through the system equilibrium condition of Equation (18).

As suggested by Gülkan and Alemdar [67], the homogeneous solution to Equation (39) can be written in the general form as

(40) $$N_{L}^{sur}\left(x\right)=\phi_{1}\left(x\right)c_{1}+\phi_{2}\left(x\right)c_{2}+\phi_{3}\left(x\right)c_{3}+\phi_{4}\left(x\right)c_{4}$$

where $c_{1}$, $c_{2}$, $c_{3}$, and $c_{4}$ are constants of integration and can be determined from the imposed boundary conditions; and $\phi_{1}$, $\phi_{2}$, $\phi_{3}$, and $\phi_{4}$ are the basic functions which forms are governed by the system parameters (see Appendix A).

To analytically determine the axial-force solution, essential and natural boundary conditions are both required. Investigating the first boundary term of Equation (29) reveals the following classical essential and natural boundary conditions

(41) $$\left.\begin{array}{l} \mathrm{specify}\;{\bar{u}}_{x}=\frac{1}{k_{S}}\left(-\frac{\partial^{2}N_{H}\left(x\right)}{\partial x^{2}}+\frac{\partial N_{L}^{sur}\left(x\right)}{\partial x}+p_{x}\left(x\right)\right)\\ \mathrm{specify}\;\bar{N}=N_{L}^{sur}\left(x\right)-\frac{\partial N_{H}\left(x\right)}{\partial x} \end{array}\right\}\mathrm{at}\;x=0,L$$

Similarly, considering the second boundary term of Equation (29) reveals the following non-classical essential and natural boundary conditions

(42) $$\left.\begin{array}{l} \mathrm{specify}\;{\bar{u}}_{x}^{’}=\frac{\partial u_{x}\left(x\right)}{\partial x}=\frac{1}{k_{S}}\left(-\frac{\partial^{3}N_{H}\left(x\right)}{\partial x^{3}}+\frac{\partial^{2}N_{L}^{sur}\left(x\right)}{\partial x^{2}}+\frac{\partial p_{x}\left(x\right)}{\partial x}\right)\\ \mathrm{specify}\;{\bar{N}}_{H}=N_{H}\left(x\right) \end{array}\right\}\mathrm{at}\;x=0,L$$

Unfortunately, boundary conditions of Equations (41) and (42) cannot be readily employed since the lower-order composite axial force $N_{L}^{sur}\left(x\right)$ is only the single variable present in the governing differential compatibility equation of Equation (39). However, boundary conditions of Equations (41) and (42) can be expressed in terms of the lower-order composite axial force $N_{L}^{sur}\left(x\right)$ by employing the axial-force relations of Equation (21). Consequently, Equations (41) and (42) become

(43) $$\left.\begin{array}{l} \mathrm{specify}\;{\bar{u}}_{x}=\frac{1}{k_{S}}\left(-\frac{l_{x}{}^{2}E_{xx}A}{{\left(EA\right)}_{xx}^{sur}}\frac{\partial^{3}N_{L}^{sur}\left(x\right)}{\partial x^{3}}+\frac{\partial N_{L}^{sur}\left(x\right)}{\partial x}+p_{x}\left(x\right)\right)\\ \mathrm{specify}\;\bar{N}=N_{L}^{sur}\left(x\right)-\frac{l_{x}{}^{2}E_{xx}A}{{\left(EA\right)}_{xx}^{sur}}\frac{\partial^{2}N_{L}^{sur}\left(x\right)}{\partial x^{2}} \end{array}\right\}\mathrm{at}x=0,L$$

(44) $$\left.\begin{array}{l} \mathrm{specify}\;{\bar{u}}_{x}^{’}=\frac{\partial u_{x}\left(x\right)}{\partial x}=\frac{1}{k_{S}}\left(\begin{array}{l} -\frac{l_{x}{}^{2}E_{xx}A}{{\left(EA\right)}_{xx}^{sur}}\frac{\partial^{4}N_{L}^{sur}\left(x\right)}{\partial x^{4}}+\\ \;\frac{\partial^{2}N_{L}^{sur}\left(x\right)}{\partial x^{2}}+\frac{\partial p_{x}\left(x\right)}{\partial x} \end{array}\right)\\ \mathrm{specify}\;{\bar{N}}_{H}=\frac{l_{x}{}^{2}E_{xx}A}{{\left(EA\right)}_{xx}^{sur}}\frac{\partial N_{L}^{sur}\left(x\right)}{\partial x} \end{array}\right\}\mathrm{at}\;x=0,L$$

## 6. Numerical Example

**Figure 5:**
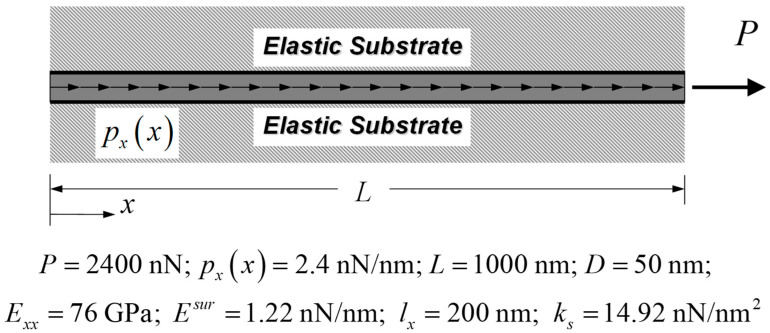
Nanowire-elastic substrate system under axial force loadings: Numerical example.

In the present study, a nanowire-elastic substrate system of Figure 5 is employed as a numerical example to investigate the characteristics and to assess the accuracy of the proposed nanobar-substrate model. An end force P of 2400 nN and a uniformly distributed load $p_{x0}$ of 2.4 nN/nm are exerted on this bar-substrate system. It is noted that this numerical example had been employed to present the characteristics of Eringen’s nonlocal bar-substrate model proposed by Limkatanyu et al. [63]. The nanowire is made of silver material with the bulk modulus $E_{xx}$ = 76 GPa and has the following geometric properties: diameter D = 50 nm and length L = 1000 nm. These mechanical and geometric properties follow values given by Juntarasaid et al. [68] and He and Lilley [48], respectively. The material length-scale parameter $l_{x}$ = 200 nm is assumed as provided by Yang and Lim [69]. As suggested by He and Lilley [48], the surface elastic modulus $E^{sur}$ of 1.22 nN/nm is employed. A stiffness coefficient $K_{S}$ of 95 × 10^−3^ nN/nm^3^ is employed for the surrounding substrate medium, thus resulting in an elastic substrate stiffness $k_{S}$ of 14.92 nN/nm^2^. This particular value for the surrounding substrate is provided by Liew et al. [70] to represent the surrounding substrate medium as polymer.

Two different bar-substrate systems are employed to simulate the responses of the silver nanowire-substrate system of Figure 5. The first is based on the classical (local) bar-substrate model [71], thus excluding both nonlocal and surface-energy effects while the second is based on the proposed strain-gradient bar-substrate model, thus including both nonlocal and surface-energy effects. Furthermore, the responses of the silver nanowire-substrate system are also simulated by the strain-gradient bar-substrate model of Sae-Long et al. [44] to confirm the accuracy and efficiency of the proposed bar-substrate model. It is worth mentioning that the strain-gradient bar-substrate model of Sae-Long et al. [44] provides the axial displacement $u_{x}^{}\left(x\right)$ as the basic solution while the proposed strain-gradient bar-substrate model yields the lower-order composite axial force $N_{L}^{sur}\left(x\right)$ as the basic solution. Unlike the strain-gradient bar-substrate model of Sae-Long et al. [44], the proposed strain-gradient bar-substrate model does not require the particular solution for the present case of the uniformly distributed load $p_{x0}$, thus showing the merit of the present model formulation.

**Figure 6:**
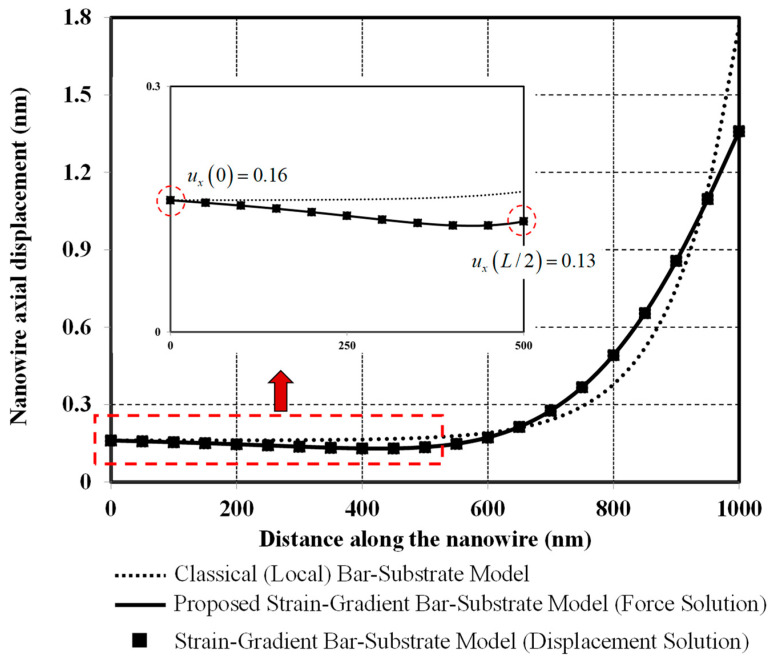
Axial displacement versus distance along the nanowire.

Figure 6 plots and compares axial-displacement profiles obtained from classical and two bar-substrate models. Clearly, the axial-displacement profile obtained from the proposed bar-substrate model is identical to that obtained from the bar-substrate model of Sae-Long et al. [44], thus confirming validity of the proposed model. Compared with the classical model, a stiffer nanowire-substrate system response is obtained with the proposed model. Considering the coefficient of the lower-order derivative (second order) term in Equation (39), the system stiffness enhancement related to the surface-free energy can clearly be noticed. However, this stiffening effect of the surface-free energy is minimal for specific values of system parameters herein since the composite bar axial stiffness ${\left(EA\right)}_{xx}^{sur}$ increases merely 0.128% with inclusion of the surface-energy effect. Therefore, the stiffening effect associated with the bar-bulk nonlocality is much more pronounced than that associated with the surface-free energy. With the classical model, the axial displacement remains approximately constant at 0.16 nm along half of the nanowire (see the inset in Figure 6) and then drastically increases to reach its maximum value of 1.8 nm at the loading end. With the proposed model, the left half of the nanowire experiences a gradual decrease in the axial displacement between its free end (0.16 nm) and its middle region (0.13 nm) while the right half of the nanowire also encounters a drastic increase in axial displacement between its middle region and its loading end (1.36 nm). This particular displacement characteristic is associated with the higher-order derivative (fourth order) term in Equation (39) and the statically indeterminate nature of the bar-substrate system.

**Figure 7:**
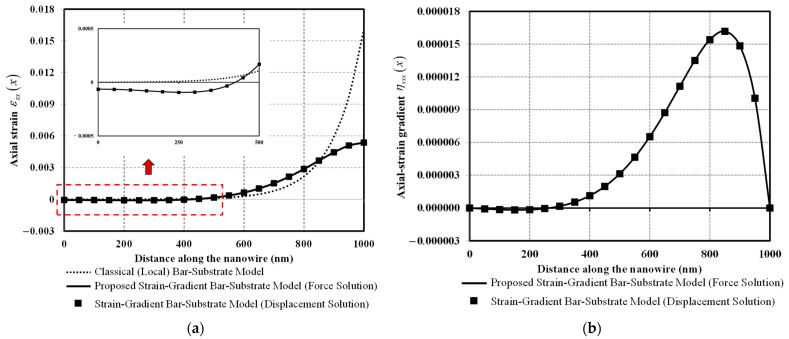
Axial strain and axial-strain gradient versus distance along the nanowire: (**a**) Axial strain; (**b**) Axial-strain gradient.

Figure 7a compares axial-strain distributions obtained from classical and two bar-substrate models while Figure 7b plots the axial-strain gradient distribution obtained from the bar-substrate models. It is clear from Figure 7a that the strain-gradient nature of the bar-bulk material drastically alters the distribution characteristics of axial-strain responses. With the classical model, the axial strain remains approximately zero along half of the nanowire (see the inset in Figure 7a) and then rapidly increases toward the loading end. In other words, the axial-strain distribution of the classical model appears to be localized in the neighborhood of the loading end. With the proposed model, the axial strain along approximately half of the nanowire is in compression (negative value) and then smoothly increases to reach its maximum positive value at the loading end. The maximum axial strain obtained with the proposed model is about three times less than that obtained with the classical model. This peculiar but unique axial-strain response complies with the axial-displacement response presented in Figure 6. The axial-strain gradient distribution is shown in Figure 7b. Vanishing of the axial-strain gradient at either nanowire end ($\eta_{xxx}^{}\left(0\right)=\eta_{xxx}^{}\left(L\right)=0$) is associated with the imposed higher-order force boundary conditions at both the nanowire ends ($N_{H}\left(0\right)=N_{H}\left(L\right)=0$) through the constitutive relation of Equation (20).

**Figure 8:**
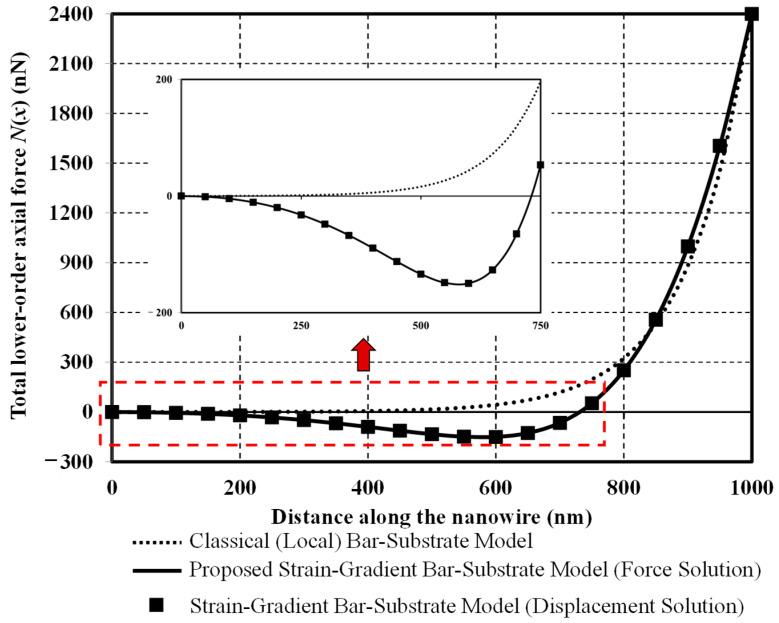
Total lower-order axial force versus distance along the nanowire.

Figure 8 compares the axial-force distributions obtained from classical and two bar-substrate models. With the classical model, the axial force remains approximately zero along the left half of the nanowire (see the inset in Figure 8) and then drastically increases toward the loading end, thus implying that only the right half of the nanowire takes part in the axial-force resistance. With the proposed model, a whole portion of the nanowire participates in the axial-force resistance. The axial force is in compression (negative value) along around three quarters of the whole length (see the inset in Figure 8) and drastically increases to reach its maximum in tension at the loading end. This unique but rather peculiar axial-force response is induced by the higher-order axial force solution to the governing differential equation of Equation (39) and the statical indeterminacy inherent to the nanowire-elastic substrate system.

**Figure 9:**
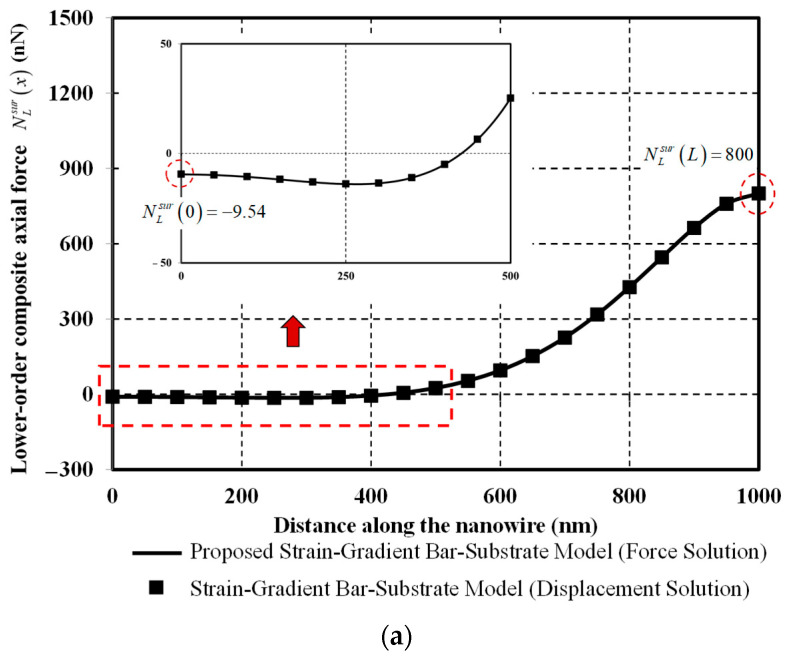

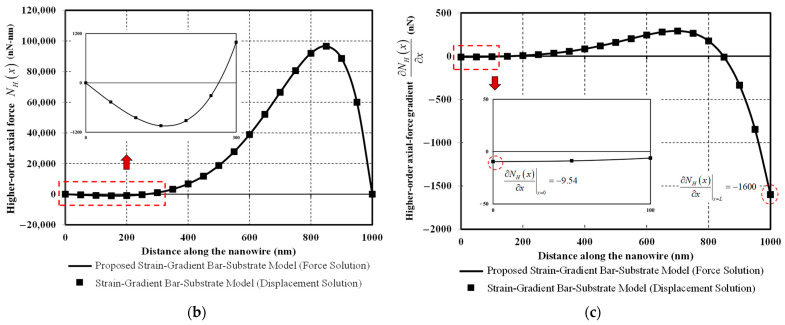
Axial force responses versus distance along the nanowire: (**a**) Lower-order composite axial force $N_{L}^{sur}\left(x\right)$; (**b**) Higher-order axial force $N_{H}\left(x\right)$; (**c**) Higher-order axial-force gradient $\partial N_{H}\left(x\right)/\partial x$.

To scrutinize the axial-force distribution nature of the proposed bar-substrate model, distribution diagrams of lower-order composite axial force $N_{L}^{sur}\left(x\right)$, higher-order axial force $N_{H}\left(x\right)$, and higher-order axial-force gradient $\partial N_{H}\left(x\right)/\partial x$ are respectively plotted in Figure 9a–c. All axial-force diagrams obtained from the bar-substrate model of Sae-Long et al. [44] are also superimposed to confirm validity of the proposed bar-substrate model. 

Figure 9a clearly shows that the lower-order composite axial force $N_{L}^{sur}\left(x\right)$ does not satisfy the end-force boundary conditions, and its distribution nature is much smoother than that of its local counterpart present in the classical model presented in Figure 8. It is worth remarking that the lower-order composite axial force $N_{L}^{sur}\left(x\right)$ is the fundamental solution to the fourth order differential equation of Equation (39) while its local counterpart is the fundamental solution to the second order differential equation. The higher-order axial force $N_{H}\left(x\right)$ is related to the derivative of the lower-order composite axial force $N_{L}^{sur}\left(x\right)$ through Equation (36).

Figure 9b shows the higher-order axial-force distribution and confirms satisfaction of the imposed higher-order end-force boundary conditions ($N_{H}\left(0\right)=N_{H}\left(L\right)=0$). It is observed that the distribution nature of the higher-order axial force is much more rapid than that of the lower-order composite axial force due to the differentiation relation between these two axial-force quantities in Equation (36).

Figure 9c presents the distribution diagram of the higher-order axial-force gradient. As indicated in Equation (30), the lower-order composite axial force $N_{L}^{sur}\left(x\right)$ and the higher-order axial-force gradient $\partial N_{H}\left(x\right)/\partial x$ both contribute to the total lower-order axial force $N\left(x\right)$. Therefore, the end-value combinations of axial-force diagrams in Figure 9a,c satisfy the end-force boundary conditions imposed on the total lower-order axial force $N\left(x\right)$ as shown in Figure 8 ($N\left(0\right)$ = 0 and $N\left(L\right)$ = 2400 nN). Furthermore, it is observed from Figure 9a,c that the higher-order axial-force gradient $\partial N_{H}\left(x\right)/\partial x$ participates more in contributing to the total lower-order axial force $N\left(x\right)$ for the present nanowire-elastic substrate system.

**Figure 10:**
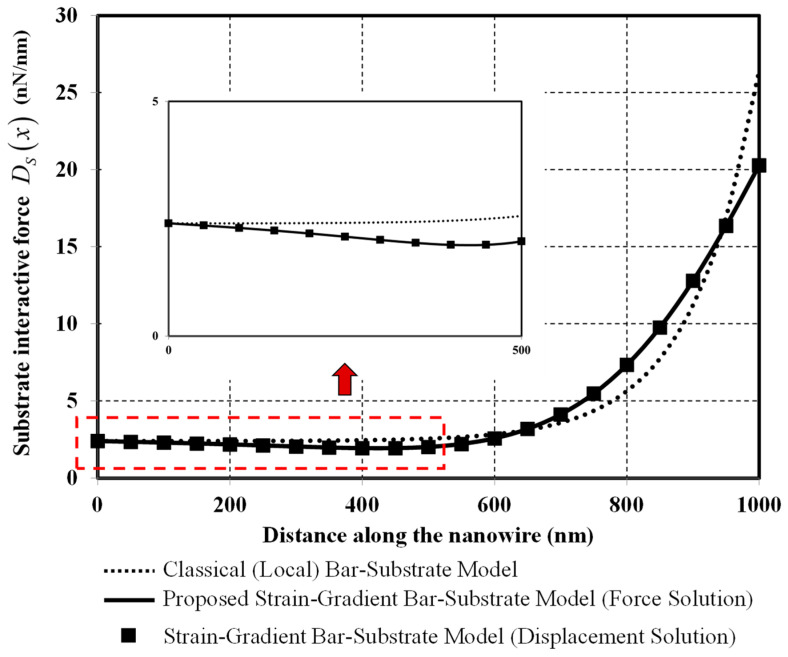
Substrate interactive force versus distance along the nanowire.

The substrate interactive-force diagrams obtained from classical and two bar-substrate models are shown in Figure 10. Complying with the Winkler-foundation hypothesis, the shapes of the substrate interactive-force diagrams resemble those of the axial-displacement diagrams shown in Figure 6. With the classical model, the substrate interactive force remains approximately constant at the value of the uniformly distributed load $p_{x0}$ of 2.4 nN/nm along half of the nanowire (see the inset in Figure 10). This observation has the physical interpretation as the bar component has no contribution to the system resistance to externally applied loads along the left half of the nanowire, thus complying with the axial-force distribution presented in Figure 8. With the proposed model, the substrate interactive force continuously varies along the length of the nanowire, thus implying the whole part of the bar component participates in the system resistance to the externally applied loads.

## 7. Summary and Conclusions

In the present work, a rational bar-elastic substrate model with inclusion of small-scale and surface-energy effects is alternatively formulated within the framework of virtual force principle. The small-scale (nonlocal) effect of the bar bulk is introduced through the thermodynamics-based strain-gradient model while the surface-energy-dependent size effect is included using the Gurtin–Murdoch surface model. To account for the bar-elastic substrate interaction, Winkler foundation model is called for. The higher-order differential compatibility equation of the problem and its associated classical and non-classical end-displacement compatibility conditions are consistently derived from the virtual force principle and form the core of the proposed bar-elastic substrate model. The axial force field serves as a basic solution to the higher-order differential compatibility equation. 

To show the accuracy and merit of the proposed bar-elastic substrate model, a nanowire-elastic substrate system under axial loadings is employed as a numerical example. Under a uniformly distributed loading, the proposed model requires no particular solution. This is in opposition to its counterpart proposed by Sae-Long et al. [44]. Considering the small-scale and surface-energy effects consistently leads to a stiffer bar-elastic substrate system in similar way as enhancement of the bar axial rigidity when compared to the classical bar-elastic substrate model. This system stiffness enhancement has been confirmed by both theoretical studies and experimental evidence available in the literature [72]. Peculiar but specific response distributions along the nanowire length are observed at both global and local levels and they are associated with the higher-order governing differential equation and the statistical indeterminacy of the nanowire-elastic substrate system. It is anticipated that the bar-elastic substrate model proposed herein will be especially useful to scientists and engineers working in the area of nanoscience and nanoengineering.

## Data Availability

The data presented in this study are available on request from the corresponding authors.

## Appendix A

The general form of the homogeneous solution to Equation (39) can be written as

(A1) $$N_{L}^{sur}\left(x\right)=\phi_{1}\left(x\right)c_{1}+\phi_{2}\left(x\right)c_{2}+\phi_{3}\left(x\right)c_{3}+\phi_{4}\left(x\right)c_{4}$$

The homogeneous solution $N_{L}^{sur}\left(x\right)$ in Equation (A1) is derived stem from the analytical solution of the beam on a two-parameter foundation as introduced by Gülkan and Alemder [67]. Thus, the basic functions in each solution cases can be expressed as:

**Case I**: $\lambda_{2}<2\sqrt{\lambda_{1}}$

(A2) $$\begin{array}{l} \phi_{1}=\cosh\left[\alpha x\right]\cos\left[\beta x\right];\;\;\phi_{2}=\sinh\left[\alpha x\right]\cos\left[\beta x\right]\\ \phi_{3}=\cosh\left[\alpha x\right]\sin\left[\beta x\right];\;\;\phi_{4}=\sinh\left[\alpha x\right]\sin\left[\beta x\right] \end{array}$$

**Case II**: $\lambda_{2}>2\sqrt{\lambda_{1}}$

(A3) $$\begin{array}{l} \phi_{1}=\cosh\left[\alpha x\right]\cosh\left[\beta x\right];\;\phi_{2}=\sinh\left[\alpha x\right]\cosh\left[\beta x\right]\\ \phi_{3}=\cosh\left[\alpha x\right]\sinh\left[\beta x\right];\;\phi_{4}=\sinh\left[\alpha x\right]\sinh\left[\beta x\right] \end{array}$$

**Case III**: $\lambda_{2}=2\sqrt{\lambda_{1}}$

(A4) $$\phi_{1}=e^{\sqrt[{4}]{\lambda_{1}}x};\;\phi_{2}=xe^{\sqrt[{4}]{\lambda_{1}}x};\;\phi_{3}=e^{-\sqrt[{4}]{\lambda_{1}}x};\;\phi_{4}=xe^{-\sqrt[{4}]{\lambda_{1}}x}$$

with the auxiliary variables as

(A5) $$\begin{array}{l} \lambda_{1}=\frac{k_{S}}{\left(l_{x}{}^{2}E_{xx}A\right)};\;\lambda_{2}=\frac{{\left(EA\right)}_{xx}^{sur}}{\left(l_{x}{}^{2}E_{xx}A\right)};\;\alpha =\sqrt{\frac{\sqrt{\lambda_{1}}}{2}+\frac{\lambda_{2}}{4}};\;\\ \beta =\sqrt{\frac{\sqrt{\lambda_{1}}}{2}-\frac{\lambda_{2}}{4}}\;\mathrm{for}\;\mathrm{Case}\;I;\;\mathrm{and}\;\beta =\sqrt{\frac{\lambda_{2}}{4}-\frac{\sqrt{\lambda_{1}}}{2}}\;\mathrm{for}\;\mathrm{Case}\;\mathrm{II} \end{array}$$
